# Rivaroxaban Treatment for Warfarin-Refractory Thrombosis in a Patient with Hereditary Protein S Deficiency

**DOI:** 10.1155/2018/5217301

**Published:** 2018-01-23

**Authors:** Koken Ameku, Mariko Higa

**Affiliations:** Department of Respiratory Medicine, Okinawa Prefectural Nanbu Medical Center & Children's Medical Center, Okinawa, Japan

## Abstract

Protein S (PS) deficiency, an autosomal dominant hereditary thrombophilia, is more prevalent in East Asian populations than in Caucasians. PS-deficient patients have historically been administered a heparin product followed by warfarin for the treatment and secondary prevention of venous thromboembolism (VTE). However, warfarin can be ineffective or causes detrimental effects in rare cases. While direct oral anticoagulants (DOACs) are being increasingly used for the treatment and prevention of VTE, their efficacy in PS-deficient patients has not been established. We describe a 91-year-old woman who presented with chronic bilateral lower leg swelling with VTE that was refractory to warfarin anticoagulation therapy for over 1 year. Her recurrent VTE was diagnosed as quantitative hereditary PS deficiency. Rivaroxaban was administered as maintenance therapy instead of warfarin; after 8 weeks, the severities of the patient's leg swelling and venous ulcerations were significantly reduced with rivaroxaban compared to warfarin, thus demonstrating the efficacy of rivaroxaban for warfarin-refractory chronic VTE associated with hereditary PS deficiency. This case illustrates that rivaroxaban can potentially serve as therapeutic agents to treat warfarin-refractory VTE in PS-deficient patients. Further investigations are required to confirm the efficacy of rivaroxaban on the long term in this regard.

## 1. Introduction

Venous thromboembolism (VTE) develops from interactions between multiple genetic and environmental risk factors [1]. Hereditary thrombophilia that is common in East Asian populations involves dysfunction of the activated protein C anticoagulant system caused by protein S (PS) and protein C (PC) deficiencies [2]. The prevalence of PS deficiency is 0.03–0.13% among Caucasians, whereas it is 1-2% in the Japanese population [3]. PS and PC are vitamin K-dependent glycoproteins that act as natural anticoagulants. PS-deficient patients have historically been administered a heparin product, followed by warfarin, for the treatment and secondary prevention of VTE. Warfarin not only functions as an anticoagulant by inhibiting vitamin K-dependent procoagulant factors (II, VII, IX, and X) but also inhibits the body's own production of the vitamin K-dependent natural anticoagulants, PS and PC. Since, in PS-deficient patients, warfarin can further decrease these natural anticoagulants, PS and PC, this may in turn offset the anticoagulant effects of warfarin to exacerbate clotting or even precipitate new clots in certain cases. Since direct oral anticoagulants (DOACs) act by directly inhibiting thrombin or factor Xa without affecting the synthesis of PS and PC, and because they have fewer drug-drug, drug-food, and drug-disease interactions compared to warfarin, they can be feasible alternatives for the treatment of warfarin-refractory patients [4]. In this case report, we describe the successful use of rivaroxaban in the treatment of a 91-year-old woman with chronic bilateral lower leg swelling and recurrent warfarin-refractory VTE with hereditary PS deficiency.

## 2. Case Presentation

**Figure 1:**
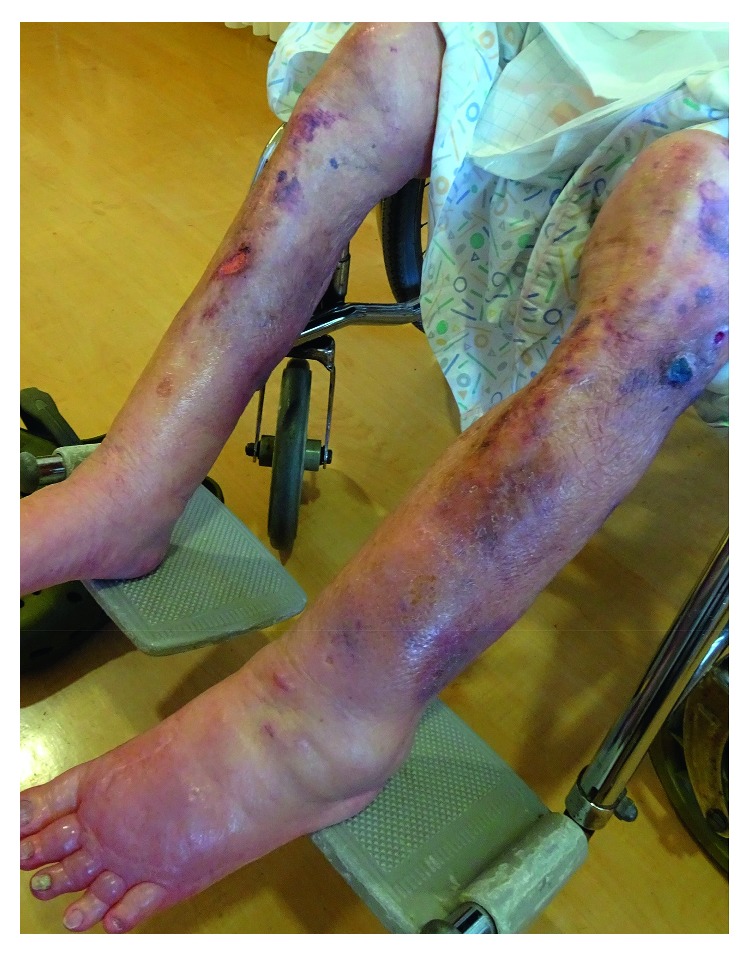
Chronic deep venous disease of the lower extremities. Leg swelling and venous ulcerations remained but were reduced in their severity after rivaroxaban therapy.

A 91-year-old woman experienced chronic bilateral lower leg swelling with VTE. She was admitted to the hospital with swollen lower legs with exudate and venous ulcerations that were diagnosed as chronic lower extremity deep venous disease. Before admission to the current hospital, she had previously undergone repeated hospitalization elsewhere for recurrent leg swelling with VTE 4 times within 1 year, for a total of 6 months. During each hospitalization, she was extensively treated with anticoagulation therapy and had shown temporary improvement. While the bilateral/unilateral leg edema and thrombosis had improved on heparin treatment, it recurred repeatedly under warfarin anticoagulation maintenance therapy after discharge. Furthermore, the patient had a medical history of subtotal gastrectomy, cholecystectomy, and chronic kidney disease (CKD), but had no history of miscarriage, thrombosis during pregnancy, or venous thrombosis until the age of 90 years. The patient's lead surgeon from her previous hospital had reported that her prothrombin time-international normalized ratio (PT-INR) was unstable owing to CKD. Current laboratory tests revealed decreased renal function, proteinuria (creatinine (Cre): 1.69 mg/dL; urine protein: 1-2 g/g Cre), and elevated levels of D-dimer (12.4 *µ*g/mL), which is a coagulation and fibrinolysis marker. PT-INR values measured on different days during treatment with 1 mg/day warfarin were 1.56, 2.39, and 4.31; they did not stabilize because of the patient's low food intake and antibiotic use for a urinary tract infection. Warfarin therapy was temporarily suspended; 2 weeks afterwards, plasma PC and PS activities were 40% (reference range, 64–146%) and <10% (reference range, 56–126%), respectively (Table 1). A PC or PS deficiency was suspected, and she was administered unfractionated heparin without warfarin. Decreased plasma levels of both total PS (52%; reference range, 65–135%) and free PS (25%; reference range, 60–150%) antigens were noted after 4 weeks of warfarin cessation and vitamin K supplementation, along with normal levels of other vitamin K-dependent coagulation proteins (factors II, VII, IX, X, and PC) (Table 1). Five weeks after warfarin cessation, decreased levels of total PS (54%) and free PS (30%) were maintained (Table 1). Anticardiolipin antibodies and lupus anticoagulant tests yielded negative results. The patient's 2 daughters, aged 66 and 54 years, also showed decreased levels of total PS (62% and 53%, resp.) and free PS (29% and 25%, resp.) antigens; furthermore, the patient's older sister had a history of refractory VTE (Table 1). Repeated VTE observed in the patient was diagnosed as quantitative hereditary PS deficiency. Intravenous administration of unfractionated heparin was continued for 7 weeks with a small amount of a diuretic (spironolactone 25 mg/day) and 10 mg/day prednisolone for chronic glomerulonephritis with proteinuria, upon which the leg swelling completely resolved and renal function was slightly improved (Cre: 1.0 mg/dL). As her past clinical course suggested the ineffectiveness of warfarin, rivaroxaban was administered as maintenance therapy (15 mg/day, which is the approved dose in Japan for long-term treatment and prevention of VTE) for 8 weeks. The patient was followed during 8 weeks of treatment; her condition improved without any major side effects. While her leg swelling and venous ulcerations were not completely cured, the reduction in their severity compared to that under warfarin therapy was notable (Figure 1).

## 3. Discussion

This patient's course revealed 3 important clinical findings. First, the DOAC rivaroxaban was effective for the treatment of warfarin-refractory chronic VTE associated with hereditary PS deficiency. Warfarin is commonly used for the long-term treatment and prevention of VTE associated with PS deficiency. However, warfarin decreases the body's production of the natural anticoagulant PS owing to its vitamin K dependency. Additionally, thrombosis may worsen in some patients with PS deficiency, as also occurs in cases of warfarin-induced skin necrosis [5]. As rivaroxaban does not decrease the production of PS, it can be more effective in cases of PS deficiency [6]. In fact, rivaroxaban did not decrease the levels of total and free PS antigen at 2 and 3 weeks of administration in our patient. Rivaroxaban had comparable efficacy and better safety when compared to warfarin in the treatment and prevention of VTE in phase 3 trial [7]. However, as patients with inherited thrombophilia presenting with VTE were not distinguished in the trials, data regarding the efficacy of DOACs for PS deficiency are limited. Additionally, the efficacy of DOACs for inherited thrombophilia is not clear and their use remains controversial [8], although some reports of positive efficacy have been published [9]. Our patient provides an important example regarding the efficacy of rivaroxaban.

Second, VTE due to PS deficiency in our patient was refractory to warfarin therapy; however, as mentioned above, warfarin may not be the best anticoagulant for patients with PS deficiency. The patient showed decreased PC and PS activities even 2 weeks after warfarin cessation. Decreased levels of PC and PS activities as a result of vitamin K suppression by warfarin might have contributed to hypercoagulation in the patient. Several incidents of ineffective treatment or detrimental effects with warfarin have been reported in patients with PS deficiency [10–12]. Moreover, it is often difficult to achieve and maintain the target PT-INR using warfarin, especially in the elderly, because of its variable response, higher sensitivity, association with comorbidities, and interactions with concomitant medications. Therefore, when using warfarin in the elderly, lower doses and more intensive monitoring are required [13, 14]. As DOACs do not decrease PS and PC and have fewer drug-drug, drug-food, and drug-disease interactions compared to warfarin, they might have a therapeutic advantage over warfarin for the treatment of patients with PS or PC deficiency [4, 6, 8].

Third, our patient had familial quantitative heterozygous PS deficiency that produced a mild phenotype. This highlights the existence of a mild form of PS deficiency that may not manifest with a thrombotic event until a well-advanced age. Neither of the patient's two daughters who had decreased levels of total and free PS antigens, the older of whom was 66 years, had developed thrombotic events. It is previously reported that 50% of patients with heterozygous PS deficiency develop VTE by the age of 55 years [3]. In contrast, only 38% of relatives of patients with PS deficiency who do not exhibit additional thrombotic deficiencies or defects (such as factor V Leiden, prothrombin G20210A, or hyperhomocysteinemia) develop VTE by the age of 65 years [1]. Even in hereditary thrombophilia, it is thought that VTE does not develop solely because of genetic risk factors. As such, our patient's VTE may have partially been attributed to additional acquired environmental risk factors or to old age, CKD, and proteinuria.

In the Japanese population, the most prevalent genetic risk factor for VTE is identified as qualitative PS deficiency caused by the K196E mutation within the *PROS1* gene (PS Tokushima) [2, 15]; approximately 1 in 58 Japanese individuals is a heterozygous carrier (1.72%). Despite the high prevalence of PS Tokushima among Japanese individuals, our patient's family likely harbors other genetic mutations that manifest as quantitative PS deficiency [16]. We did not perform genetic analysis on our patient, as the technique was not readily available. An enzyme-linked immunosorbent assay (ELISA) system using the PS K196E mutation-specific antibody is now being developed for rapid identification of PS Tokushima carriers and will be available in the near future [17].

The efficacy and safety of DOACs in patients who are elderly, have renal impairment, or have low body weight are important to note, as all the DOACs are dependent on renal clearance to varying extents [18]. These patient subgroups have elevated risks of both embolic and bleeding events. Even in these subgroups, anticoagulant therapy has antithrombotic benefits that outweigh the risk of bleeding [13]. In so far as using rivaroxaban for VTE treatment, pooled and subgroup analyses of the phase III EINSTEIN DVT [7] and EINSTEIN PE [19] studies were conducted. In the pooled analysis, similar efficacy (hazard ratio (HR): 0.68), a significantly lower rate of major bleeding (HR: 0.27), and a significantly more favorable net clinical benefit (HR: 0.51) were reported in fragile patients who were over 75 years, or creatinine clearances (CrCl) < 50 mL/min or body weights < 50 kg [20]. In the prespecified subgroup analysis, although both VTE recurrence and major bleeding events increased according to the severity of renal impairment following enoxaparin/vitamin K antagonist (VKA) treatment, only increased VTE recurrence with similar efficacy to enoxaparin/VKA was observed using rivaroxaban treatment. However, major bleeding was not increased by renal impairment and occurred less frequently compared with enoxaparin/VKA (HR: 0.79 in patients with CrCl > 80 mL/min; HR: 0.44 in those with CrCl 50–79 mL/min; and HR: 0.25 in those with CrCl 30–49 mL/min) [21]. Of note, patients with severe renal impairment (CrCl < 30 mL/min) were excluded from these 2 studies. Furthermore, noninferior efficacy and a lower incidence of primary adverse events were shown when using both apixaban and edoxaban compared to warfarin in the elderly [13, 22]. Our patient was 91 years old with a CrCl of 30 mL/min and a low body weight of 35 kg. Among the 3 DOACs (rivaroxaban, apixaban, and edoxaban) currently available to treat VTE in Japan, we chose rivaroxaban because it better assured compliance among the elderly as it is administered once daily (compared to apixaban which is administered twice daily) and has a relatively smaller fraction of renal clearance as an unchanged drug (36%) than edoxaban (50%). Plasma rivaroxaban concentration-time profiles for patients of extremely old age (90 years), renal function (CrCl: ∼30 mL/min), and body weight (∼45 kg) were simulated using pharmacokinetic data collected from the 2 phase II studies of rivaroxaban for the treatment of acute deep vein thrombosis. The *C*_max_ values in all simulations were within the 5th to 95th percentile ranges of the population's mean values, showing that age and renal function had a moderate influence on rivaroxaban exposure and that the influence of body weight was small. Thus, fixed dosing of rivaroxaban was considered feasible for a broad segment of the patient population [23]. These predictable pharmacokinetic profiles could be associated with the efficacy and safety of rivaroxaban as shown in the phase III studies, and also in our patient. Owing to the different regimens used for VTE treatment in Japan (low-molecular-weight heparins are not used, and the PT-INR target when administering warfarin is 1.5–2.5), Japanese patients were not enrolled in the global EINSTEIN DVT and PE trials. Instead, the domestic J-EINSTEIN DVT and PE programs were performed and, as with the 2 global trials, showed similar efficacy and safety with rivaroxaban [24]. In Japan, the approved dose of rivaroxaban for VTE is 15 mg twice daily for 3 weeks followed by 15 mg once daily. Anticoagulation therapy requires long-term administration for patients with inherited thrombophilia. Rivaroxaban therapy was also demonstrated to be safe when administered for as long as 1 year in the EINSTEIN EXTENSION [7] and EINSTEIN CHOICE [25] studies. Overall, data for elderly patients, those with renal impairment, those with low body weight, and the effect of extended-term treatment remain scarce; hence, additional research is required in the real-world setting.

In conclusion, our patient demonstrated the efficacy of rivaroxaban for warfarin-refractory chronic VTE associated with hereditary PS deficiency. DOACs may therefore be therapeutic alternatives for warfarin-refractory VTE in PS-deficient patients. Further investigations are required to confirm the efficacy of rivaroxaban on the long term in this patient population.

## Figures and Tables

**Table 1:** Coagulation studies of the patient and her two daughters.

		Normal range	Patient (91 years)					Daughter 1 (66 years)	Daughter 2 (54 years)
			Period after warfarin cessation			Period after rivaroxaban			
			2 weeks	4 weeks	5 weeks	2 weeks	3 weeks		
Total PS antigen	%	65–135	—	52	54	57	54	62	53
Free PS antigen	%	60–150	—	25	30	27	27	29	25
PS activity	%	56–126	<10	—	—	—	—	—	—
PC activity	%	64–146	40	120	—	—	—	156	131
PT-INR			1.11	0.96	1.00	0.89	0.85	1.02	1.10
II	%	75–135	—	76	—	—	—	—	—
VII	%	75–140	—	146	—	—	—	—	—
IX	%	70–130	—	105	—	—	—	—	—
X	%	70–130	—	85	—	—	—	—	—

PS: protein S; PC: protein C; PT-INR: prothrombin time-international normalized ratio.

## References

[1] Brouwer J. L., Veeger N. J., Kluin-Nelemans H. C., van der Meer J. (2006). The pathogenesis of venous thromboembolism: evidence for multiple interrelated causes. *Annals of Internal Medicine*.

[2] Hamasaki N., Kuma H., Tsuda H. (2013). Activated protein C anticoagulant system dysfunction and thrombophilia in Asia. *Annals of Laboratory Medicine*.

[3] ten Kate M. K., van der Meer J. (2008). Protein S deficiency: a clinical perspective. *Haemophilia*.

[4] Burnett A. E., Mahan C. E., Vazquez S. R., Oertel L. B., Garcia D. A., Ansell J. (2016). Guidance for the practical management of the direct oral anticoagulants (DOACs) in VTE treatment. *Journal of Thrombosis and Thrombolysis*.

[5] Lipe B., Ornstein D. L. (2011). Deficiencies of natural anticoagulants, protein C, protein S, and antithrombin. *Circulation*.

[6] Skelley J. W., White C. W., Thomason A. R. (2017). The use of direct oral anticoagulants in inherited thrombophilia. *Journal of Thrombosis and Thrombolysis*.

[7] EINSTEIN Investigators, Bauersachs R., Berkowitz S. D., Brenner B. (2010). Oral rivaroxaban for symptomatic venous thromboembolism. *New England Journal of Medicine*.

[8] Undas A., Góralczyk T. (2016). Direct oral anticoagulants in patients with thrombophilia: challenges in diagnostic evaluation and treatment. *Advances in Clinical and Experimental Medicine*.

[9] Martinelli I., Bucciarelli P., Artoni A. (2013). Anticoagulant treatment with rivaroxaban in severe protein S deficiency. *Pediatrics*.

[10] Haran M. Z., Lichman I., Berebbi A., Weinmann E., Rosenberg N. (2007). Unbalanced protein S deficiency due to warfarin treatment as a possible cause for thrombosis. *British Journal of Haematology*.

[11] Odegaard O. R., Lindahl A. K., Try K., Kvalheim G., Hjalmar Sørbø J. (1992). Recurrent venous thrombosis during warfarin treatment related to acquired protein S deficiency. *Thrombosis Research*.

[12] Sallah S., Abdallah J. M., Gagnon G. A. (1998). Recurrent warfarin-induced skin necrosis in kindreds with protein S deficiency. *Haemostasis*.

[13] Andreotti F., Rocca B., Husted S. (2015). Antithrombotic therapy in the elderly: expert position paper of the European Society of Cardiology Working Group on Thrombosis. *European Heart Journal*.

[14] Karamichalakis N., Georgopoulos S., Vlachos K. (2016). Efficacy and safety of novel anticoagulants in the elderly. *Journal of Geriatric Cardiology*.

[15] Miyata T., Maruyama K., Banno F., Neki R. (2016). Thrombophilia in East Asian countries: are there any genetic differences in these countries?. *Thrombosis Journal*.

[16] García de Frutos P., Fuentes-Prior P., Hurtado B., Sala N. (2007). Molecular basis of protein S deficiency. *Thrombosis and Haemostasis*.

[17] Maruyama K., Akiyama M., Kokame K., Sekiya A., Morishita E., Miyata T. (2015). ELISA-based detection system for protein S K196E Mutation, a genetic risk factor for venous thromboembolism. *PLoS One*.

[18] Turpie A. G. G., Purdham D., Ciaccia A. (2017). Nonvitamin K antagonist oral anticoagulant use in patients with renal impairment. *Therapeutic Advances in Cardiovascular Disease*.

[19] EINSTEIN–PE Investigators, Büller H. R., Prins M. H., Lensin A. W. (2012). Oral rivaroxaban for the treatment of symptomatic pulmonary embolism. *New England Journal of Medicine*.

[20] Prins M. H., Lensing A. W., Bauersachs R. (2013). Oral rivaroxaban versus standard therapy for the treatment of symptomatic venous thromboembolism: a pooled analysis of the EINSTEIN-DVT and PE randomized studies. *Thrombosis Journal*.

[21] Bauersachs R. M., Lensing A. W., Prins M. H. (2014). Rivaroxaban versus enoxaparin/vitamin K antagonist therapy in patients with venous thromboembolism and renal impairment. *Thrombosis Journal*.

[22] Geldhof V., Vandenbriele C., Verhamme P., Vanassche T. (2014). Venous thromboembolism in the elderly: efficacy and safety of non-VKA oral anticoagulants. *Thrombosis Journal*.

[23] Mueck W., Stampfuss J., Kubitza D., Becka M. (2014). Clinical pharmacokinetic and pharmacodynamic profile of rivaroxaban. *Clinical Pharmacokinetics*.

[24] Yamada N., Hirayama A., Maeda H. (2015). Oral rivaroxaban for Japanese patients with symptomatic venous thromboembolism - the J-EINSTEIN DVT and PE program. *Thrombosis Journal*.

[25] Weitz J. I., Lensing A. W. A., Prins M. H. (2017). Rivaroxaban or aspirin for extended treatment of venous thromboembolism. *New England Journal of Medicine*.

